# Analysis of the Umami Taste and Volatile Flavor Components of *Lentinus edodes* Stipe Hydrolysates Derived After Different Enzymatic Treatments

**DOI:** 10.3390/foods15142495

**Published:** 2026-07-14

**Authors:** Qian Zhu, Jingjing Du, Jiayu Gu, Jiagang Guo, Yuhan Wu, Shuo Wang, Jian Jiang, Song Yang

**Affiliations:** 1 Institute of Agro-Products Processing, Anhui Academy of Agricultural Sciences, Hefei 230031, China; zqahjgs@126.com (Q.Z.); dujjmm@126.com (J.D.); gujiayu2018@126.com (J.G.); guojiagang@163.com (J.G.); wuyuhan63@163.com (Y.W.); 18291892190@163.com (S.W.); 2 Anhui Engineering Laboratory of Food Microbial Fermentation and Functional Application, Hefei 230031, China; 3 Anhui Province Vegetable Industry Technology System, Hefei 230031, China; 4 Anhui Agro-Products Processing Industrial Technology System, Hefei 230031, China

**Keywords:** degree of hydrolysis, Flavourzyme, sensory evaluation, odor activity value, by-product valorisation

## Abstract

*Lentinus edodes* stipe is an under-utilised processing by-product that holds potential as a natural umami source. This study systematically compared nine enzymatic hydrolysis combinations, including single proteases, cellulase, and double enzyme systems comprising both protease–protease and protease–cellulase mixtures, to optimise the release of taste-active and volatile flavor compounds from the stipes. The Flavourzyme-only hydrolysate achieved the highest degree of hydrolysis (50.38%), the richest 5′-nucleotides (5.46 mg/g), free amino acids (59.06 mg/g) and volatile compounds (150.23 μg/kg), and an equivalent umami concentration of 288 g monosodium glutamate equivalent per 100 g matter. It also delivered the strongest umami taste and the lowest bitterness in sensory evaluation. The double-enzyme systems consistently underperformed due to competitive substrate binding, suboptimal pH for companion enzymes, and potential mutual protease digestion. Among the volatile compounds, 1,2,4-trithiolane and cedrol were identified as key aroma differentiators by multivariate analysis. These findings demonstrate that Flavourzyme alone constitutes the optimal enzymatic system for producing clean-tasting, umami-rich condiments from *L. edodes* stipes and caution against indiscriminate enzyme blending without prior compatibility assessment. The optimized hydrolysis process provides a scalable route for the industrial valorisation of mushroom processing by-products.

## 1. Introduction

*Lentinus edodes* are grown worldwide and widely appreciated by consumers for their distinct flavor and high nutritional value [1]. China is the largest producer and consumer of *L. edodes* in the world, with a production of 1.296 × 10^7^ t in 2022, accounting for 98.3% of the total global production [2]. *L. edodes* is rich in proteins, polysaccharides and amino acids (AAs) [3]. Most importantly, developing natural flavorings from *L. edodes* has been a research hotspot because of its unique umami taste and abundance of volatile flavor substances [1]. The *L. edodes* body generally consists of a pileus and a stipe. Most studies on the umami and aroma of *L. edodes* have examined the pileus exclusively [4,5]; the stipe has received considerably less attention. Stipes constitute 25–33% of the fresh weight of *L. edodes* and are usually regarded as a by-product for low-value applications, since their relatively high fiber content results in poor taste [6,7]. Nevertheless, they retain appreciable quantities of nutrients and minerals [7]. Previous studies have demonstrated that umami ingredients obtained from *L. edodes* stipes can function as low-sodium flavor enhancers and successfully replace monosodium glutamate (MSG) in food formulations [6,8,9]. Efficient enzymatic processes can convert this abundant by-product into high-value natural flavorings. This approach offers both economic and sustainability benefits for the mushroom industry.

Enzymatic hydrolysis is a widely adopted technique for preparing mushroom flavorings because of its advantages of mild and controllable conditions and environmental sustainability [10]. Prandi et al. [11] demonstrated that enzyme-assisted extraction was the most efficient technique for protein recovery from six different mushroom by-products. Proteases cleave the macromolecular proteins of *L*. *edodes* into peptides and free amino acids (FAAs), which are important contributors to umami taste [1]. Cellulose is the major structural polysaccharide in the cell walls of *L. edodes* [5]. Cellulase can also release proteins and flavor precursors from the cell wall by breaking down structural polysaccharides, thereby helping to improve extraction efficiency. Zhu et al. [12] applied ultrasonic-assisted two-stage enzymatic hydrolysis with cellulase, pectinase, and papain to extract soluble substances from mixed edible fungi residues, achieving a 2.87-fold increase in soluble solids and a 12.19-fold improvement in umami response. However, the extremely low pectin content reported for these materials means that pectinase contributes negligibly to cell wall disruption [13]. Therefore, selecting cellulase for hydrolysis would be much more effective than pectinase in breaking down the cell walls of *L*. *edodes* stipes and releasing intracellular proteins and flavor precursors. Notably, peptides obtained from enzymatic hydrolysis containing hydrophobic groups are bitter [14]. However, extensive hydrolysis has been shown to reduce bitterness, underscoring the critical role of enzyme selection [15]. Different enzymes with various specificities and binding sites lead to products with various sensory properties and flavor characteristics [16]. It was generally assumed that enzyme mixtures produce synergistic effects. However, recent studies on fungal and plant proteins indicated that overlapping cleavage sites and unsuitable reaction conditions can cancel these advantages and even lead to competitive interference [17,18]. Thus, choosing suitable enzymes is essential for increasing hydrolysis efficiency and producing flavorful hydrolysates from *L. edodes* stipes.

To date, few systematic studies have addressed which enzymatic treatment optimally regulates the flavor quality of *L*. *edodes* stipe hydrolysates, and the volatile fraction of mushroom hydrolysates has received far less scrutiny than the non-volatile taste profile. The aims of this study are: (1) to compare the effects of different single and composite enzyme treatments on the hydrolysis efficiency of *L. edodes* stipes and the formation of taste-active and aroma-active compounds; (2) to elucidate the relationship between enzyme specificity and the generation of key flavor compounds; (3) to identify the optimal enzyme treatment options for producing the best flavor profile, thereby providing a basis for its development as a natural flavor enhancer.

## 2. Materials and Methods

### 2.1. Materials and Chemicals

*L. edodes* stipes and *L. edodes* (intact basidiomata) were obtained from Tongling Kangxin Agricultural Technology Co., Ltd. (Tongling, China). Commercial enzymes including Flavourzyme (30,000 U/g), Neutrase (200,000 U/g), Papain (100,000 U/g), Bromelain (200,000 U/g) and Cellulase (10,000 U/g) were obtained from Nanning Doing-Higherbio-tech Co., Ltd. (Nanning, China). 5′-Nucleotide standards were purchased from Yuanye Bio-Technology Co., Ltd. (Shanghai, China).

### 2.2. Chemical Analyses

Before analysis, the *L. edodes* stipes were washed, air-dried at 50 °C for 12 h, ground, and passed through a 60-mesh sieve to obtain a powder. Intact basidiomata of *L. edodes* were processed in the same manner. The method described by Li et al. [7] was used to examine the moisture, total protein, total sugar, crude fiber, ash, and fat contents of the *L. edodes* stipes.

### 2.3. Preparation of the Lentinus edodes Stipe Hydrolysates (LSHs)

The *L. edodes* stipes powder was combined with distilled water in a solid/liquid (*w*/*v*) ratio of 1:15. The mixture was then stirred (300 r/min) and equilibrated at 50 °C for 10 min using a magnetic stirrer (HH-6J, Changzhou Jintan Youlian Instrument Research Institute, Changzhou, China). Then, the mixture was adjusted to the optimum pH for the selected enzyme. The desired enzyme was added to the *L. edodes* stipes slurry at 4000 U of enzyme per gram of substrate. The single-enzyme groups included Flavourzyme (pH 7.0), Neutrase (pH 7.0), Papain (pH 7.0), Bromelain (pH 7.0), and Cellulase (pH 5.0). Additionally, the double-enzyme combinations of Flavourzyme-Neutrase (FeN) (pH 7.0), Flavourzyme-Papain (FeP) (pH 7.0), Flavourzyme-Bromelain (FeB) (pH 7.0), and Flavourzyme-Cellulase (FeC) (pH 7.0) were blended in a 1:1 (*w*/*w*) ratio. Following the addition of the enzyme, the reaction proceeded for 2 h at 50 °C with constant stirring at 300 r/min. After hydrolysis, the enzyme was immediately deactivated by heating to 100 °C for 10 min and then separated by centrifugation (H2-16KR, Kecheng Instrument Equipment Co., Ltd., Changsha, China) at 8000× *g* for 10 min at 4 °C to obtain the supernatant. The above method was repeated for the control group, which did not include an enzyme.

### 2.4. Protein Recovery (PR) and Degree of Hydrolysis (DH) Assays

The PR and DH were determined according to the methods of Gao et al. [16]. The protein content was measured via the bicinchoninic acid method, and the DH was determined via the o-phthalaldehyde method. The PRs of the LSHs were calculated as follows:

(1) $$Protein\;recovery\left(\%\right)=\frac{weight\;of\;protein\;in\;hydrolysate}{weight\;of\;protein\;in\;raw\;powder}\times 100$$

### 2.5. Color Measurement

A colorimeter (CR-400, Konica Minolta, Tokyo, Japan) was used to measure the color values of the samples. Six measurements were taken for each LSH.

### 2.6. Determination of Free Amino Acids (FAAs)

FAAs were detected according to the methods of Liu et al. [19], with slight modifications. A 5 mL of LSH was blended with 1 mL of sulfosalicylic acid (10 g/L) and 0.5 mL of EDTA-Na_2_ (10 g/L), and the resulting mixture was ultrasonicated for 1 h, incubated at 4 °C for 16 h, and then centrifuged at 5000× *g* and 4 °C for 10 min. A rotary evaporator was used to dry 5 mL of the supernatant, and the volume of the sample was subsequently fixed to 5 mL by the addition of HCl solution (0.02 mol/L). An automatic amino acid analyzer (L-8900, Hitachi, Tokyo, Japan) was used to further examine the extracted solution after it had been filtered through a 0.22 μm filter. The analyzer uses ion-exchange chromatography to separate amino acids, followed by a post-column ninhydrin reaction detection system. The detection parameters were as follows: the sample volume was 20 μL, the pump flow rate range was 0–0.9 mL/min, the column temperature was 57 °C, the reactor temperature was 135 °C, and the detection occurred at 570 nm and 440 nm (for proline). Tryptophan was not included in the calibration standard and therefore was not determined. Consequently, the total essential amino acid values reported in this work exclude tryptophan.

### 2.7. Determination of 5′-Nucleotides

Prior to high-performance liquid chromatography (HPLC) analysis (LC-20A, Shimadzu, Kyoto, Japan), each sample was filtered through a 0.22 μm filter. A methanol (0.05 mol/L) potassium dihydrogen phosphate solution (3/97, *v*/*v*) was used as the mobile phase at a flow rate of 0.3 mL/min. The external standard method was used for substance identification and quantification.

### 2.8. Taste Active Value (TAV) and Equivalent Umami Concentration (EUC) Assays

The TAVs were calculated by comparing the levels of taste compounds to their threshold values [1,14], whereas the EUC was determined as the equivalent concentration of MSG (g/100 g). The EUC was derived via the methodology of Yamaguchi et al. [20] and represents the umami intensity produced by the combination of umami AAs (Asp and Glu) and 5′-nucleotides (5′-AMP, 5′-GMP, and 5′-IMP).

### 2.9. Sensory Evaluation

Ten seasoned panelists (six females and four males aged 20–30 years) were invited to evaluate the samples, and each panelist was tested and trained before sensory evaluation began. According to Chen et al. [14], the quantitative descriptive evaluation was conducted using a 10-point scale (where 0 denotes no indication of the sensory attribute and 10 denotes the strongest). The sensory characteristics of the LSHs were examined according to the following five attributes with the following reference sample solutions: 3.5 mg/mL MSG (umami), 3.5 mg/mL sodium chloride (saltiness), 10 mg/mL sucrose (sweetness), 0.8 mg/mL citric acid (sourness), and 0.05 mg/mL quinine sulfate (bitterness). Each standard solution was set on a 5-point scale. The test samples and standard solutions were held at 40 °C and were supplied randomly. Each sample was evaluated in triplicate.

### 2.10. Determination of Volatile Compounds

Headspace solid-phase microextraction (HS-SPME) coupled with gas chromatography–mass spectrometry (GC–MS, Shimadzu, Tokyo, Japan) was used for volatile compound determination. Briefly, 20 µL of internal standard (D-chlorobenzene (1 mg/L)) was added to a headspace container containing 5 mL of sample. A headspace extraction needle (50/30 μm, 57328-U DVB/CAR/PDMS, Supelco, Bellefonte, PA, USA) was used to extract the sample for 45 min at 50 °C. The sample was then resolved in a gas chromatography injector at 250 °C for 5 min before analysis with a DB-WAX column (60 m × 250 μm × 1.4 μm, Thermo Scientific, Waltham, MA, USA) in the non-split injection mode. The chemicals were identified by mass spectrometry upon comparison with the NIST 20 library. The content of each compound was calculated by comparing the peak area of the compound with that of the internal standard. The odor activity value (OAV) of each of the volatile compounds was calculated by dividing the concentration of the compound by the odor threshold value.

### 2.11. Statistical Analysis

All tests were performed in triplicate, and the results are expressed as the means ± standard deviations. The data were statistically analyzed using SPSS 26 and plotted using Origin 2022. The differences were determined with one-way (ANOVA) followed by Duncan’s test, where *p* < 0.05 indicated significance. Partial least squares discriminant analysis (PLS-DA) was carried out with MetaboAnalyst 6.0 (https://www.metaboanalyst.ca/).

## 3. Results and Discussion

### 3.1. Contents of Basic Components of Lentinus edodes Stipes

The basic chemical composition of dehydrated stipes and intact basidiomata of *L. edodes* was determined prior to the enzymatic hydrolysis step. Table S1 shows that the crude fiber content in the stipes (26.07%) was marginally greater than that in intact *L. edodes* (22.05%), but the total protein content in the *L. edodes* stipes (15.24%) was lower than that in the intact material (21.27%). In addition, intact basidiomata and stipes of *L. edodes* showed high sugar contents of 31.64% and 32.30%, respectively. Li et al. [7] reported that *L. edodes* stipe had greater fiber and carbohydrate contents than the pileus did. Thus, it was indicated that *L. edodes* stipes contains an abundance of nutrients and could be utilized as raw materials for natural edible mushroom flavor processing. Previous studies have also revealed the presence of considerable amounts of umami compounds in *L. edodes* stipes with the potential to replace MSG in snack products [6,8]. Therefore, further research on the umami substances in LSHs is needed.

### 3.2. Protein Recovery (PR), Degree of Hydrolysis (DH), and Color Characteristics of the Lentinus edodes Stipe Hydrolysates (LSHs)

As an indicator of protein hydrolysis efficiency, the PR signifies the sensitivity of *L. edodes* stipe proteins to different biological enzymes [16]. Overall, 40.51% of the *L. edodes* stipe proteins dissolved without enzymatic treatment, whereas significantly more proteins were recovered in the LSHs (Table 1). The high protein recovery values observed in the enzyme-treated groups are consistent with the findings of Prandi et al. [11], who reported that enzyme-assisted extraction outperformed both aqueous and ultrasound-assisted methods for protein recovery from mushroom by-products. The highest PR value (62.90%) was recorded for the Cellulase single-enzyme group, which was attributed mainly to the release of proteins bound to the polysaccharides of plant tissues [21]. In contrast, Flavourzyme and Neutrase hydrolysis yielded the lowest PR values of 47.56% and 47.92%, respectively. This might be due to the strong endopeptidase and exopeptidase activities of Flavourzyme, which produce many FAAs that cannot be detected by the protein content measurement methods used [16]. The highest DH value (50.38%) in the Flavourzyme hydrolysates confirmed this hypothesis. Among the double-enzyme groups, FeC presented the highest PR (60.64%), while FeN presented the lowest (49.19%). Notably, the double-enzyme treatments did not enhance the proteolysis rate compared to single-enzyme treatments. This lack of synergy might be related to multiple factors, including the enzyme’s kinetic properties and compatibility. Substrate specificity overlap resulted in competitive rather than complementary action of enzymes at cleavage sites [18,22], while compromises in reaction conditions (such as pH and temperature) prevented both enzymes from achieving optimal activity. Therefore, the anticipated additive effect was not realized under the applied conditions.

The DH indicates the proportion of peptide bonds hydrolyzed by the enzyme. The ability to obtain protein hydrolysates with high DH values and thus reduced bitter taste is crucial in the preparation of natural flavoring matrices. The DH of the control (19.16%) was significantly lower than that of the protease-prepared LSHs (Table 1). Cellulase was ineffective in terms of protein hydrolysis (20.77%). There were also considerable differences in the DH values of various LSHs, which suggests that the different peptide cleavage patterns of the proteases significantly affected the extent of *L. edodes* stipe protein hydrolysis [23]. Among the single-enzyme groups, Flavourzyme degraded the *L. edodes* stipe macromolecular proteins and polypeptides most efficiently and gave the highest DH value (50.38%). This might be because Flavourzyme has both endopeptidase and exopeptidase activities and has a wider range of peptide bond cleavage sites [17]. Additionally, prior research has shown that Flavourzyme can effectively hydrolyze both plant and animal proteins [4,24,25]. Notably, all of the hydrolysis effects in the double-enzyme groups were weaker than those in the Flavourzyme group but greater than those in the other single-enzyme groups. Flavourzyme contributed significantly to hydrolysis at the same concentration, whereas any enzyme in combination with Flavourzyme did not provide more cleavage sites to enhance the hydrolysis of *L. edodes* stipe proteins. In contrast, previous studies reported that a combination of enzymes increased the efficiency of protein hydrolysis [16,26]. Thus, protease specificity and interactions that alter the kinetics of the proteases might be involved [17,27].

From a by-product valorisation perspective, the PR values obtained in this study demonstrate that enzymatic hydrolysis can effectively recover proteins from *L. edodes* stipes, with the Cellulase treatment achieving 62.90% and Flavourzyme 47.56%. Both the Cellulase and the FeC treatments achieved PR exceeding 60%, indicating that cellulase offers a marked advantage in protein recovery and that minimal amounts of protein remained in the insoluble residue. The lower PR observed for Flavourzyme does not imply lower valorisation efficiency. Rather, Flavourzyme produced the highest DH and the most abundant FAAs, which are the direct contributors to umami taste. By comparison, although the Cellulase and FeC treatments achieved higher PRs, they performed poorly in terms of DH. These results indicate that enzymatic hydrolysis, particularly with Flavourzyme, can convert *L. edodes* stipes into a value-added flavoring base, with approximately half of the initial protein being recovered in soluble form. The remaining insoluble fraction likely still contains protein embedded within the cell-wall matrix, presenting an opportunity for further recovery through sequential or intensified hydrolysis strategies. Adopting a sequential hydrolysis approach in future studies, in which cellulase pre-treatment is followed by Flavourzyme hydrolysis, may further improve both protein recovery and the degree of hydrolysis, thereby promoting the valorisation of edible mushroom by-products.

There were significant differences in the colors of LSHs (Table 1). Compared with those of the control, the L* and b* values of the LSHs were significantly lower, and the a* value was significantly higher, with the exception of the Cellulase group, which presented the opposite color changes. All of the enzyme hydrolysates presented a large color difference, with Cellulase having the lowest *ΔE* of 8.19. Color is a major factor in consumer acceptance. The change in color after enzymatic hydrolysis might be associated with the occurrence of the Maillard reaction upon heating or pigment dissolution in plant tissues [16,28]. The impact of color differences on the application of LSHs is application-dependent. However, it should be noted that excessively dark-colored ingredients are generally undesirable in food [6]. In this study, the Cellulase hydrolysate exhibited a relatively light color, whereas the protease hydrolysates developed a yellowish-brown color. The colors of these hydrolysates fall within the common color range of many seasonings and are therefore suitable for use in the production of flavor bases.

### 3.3. Analysis of the Taste-Active Compounds in the Lentinus edodes Stipe Hydrolysates (LSHs)

#### 3.3.1. 5′-Nucleotides Analysis

5′-Nucleotides act synergistically with MSG because of their flavor-enhancing properties [29]. The control group contained 2.83 mg/g total 5′-nucleotides, among which 5′-CMP was the major nucleotide in the nonenzymatically treated *L. edodes* stipes (Table 2). Except for the considerable drop observed for the Cellulase hydrolysate, the total amounts of 5′-nucleotides were significantly greater in the single-enzyme groups following enzymatic hydrolysis. The highest 5′-nucleotide contents were found in the Flavourzyme and Neutrase hydrolysates (5.46 and 5.50 mg/g, respectively). The 5′-nucleotide contents in the double protease hydrolysates were almost all lower than those in the single protease hydrolysates, which is in agreement with the results of Zhang et al. [28]. Additionally, the single protease groups presented significantly higher levels of 5′-AMP, 5′-GMP, 5′-IMP, and 5′-UMP than did the control group. 5′-AMP, 5′-GMP, and 5′-IMP are known as flavor nucleotides with an umami taste. 5′-GMP produces a meaty flavor and is believed to be a stronger taste enhancer than MSG, and 5′-AMP can provide sweetness and inhibit bitterness [19]. This research demonstrated that hydrolysis of *L. edodes* stipes with a single protease resulted in better umami properties, among which Flavourzyme and Neutrase performed the best.

#### 3.3.2. Free Amino Acids (FAAs) Analysis

The classification of FAAs in the LSH and control groups according to taste characteristics is shown in Table 2. Compared with those in the control group, the hydrolysates prepared with Flavourzyme, alone and in combination with other proteases, contained significantly greater amounts of each AA. The total content of FAAs increased from 25.25 mg/g in the control to 47.65–59.06 mg/g in these groups. Furthermore, the total essential amino acid (EAA) content (25.15–31.41 mg/g) notably increased. This result was consistent with the DH values, which indicated that the majority of the *L. edodes* stipe proteins decomposed into FAAs and short soluble peptides during hydrolysis in these enzyme systems. Among the LSHs, the Flavourzyme hydrolysate contained the highest concentration of FAAs, which could explain its highest DH value. The Flavourzyme hydrolysates contained significantly higher levels of both umami and sweet AAs, with total levels of 8.24 mg/g and 15.21 mg/g, respectively, which directly contributed to their superior umami intensity. This can be attributed to the unique combination of endo- and exopeptidase activities inherent to Flavourzyme [17]. The endopeptidase initially cleaves protein molecules into smaller peptides, thereby increasing the number of accessible termini for the exopeptidase (aminopeptidases and carboxypeptidases). The exopeptidase then efficiently releases large quantities of flavor-active FAAs from the N- and C-termini of peptides, particularly the key umami-giving Glu and Asp [30]. Beyond these two primary umami amino acids, other FAAs can also modulate umami perception. Zhan et al. [31] reported that Gly, Ala, Pro, Tyr, Thr, and Ile positively affect umami intensity, likely mediated through synergistic interactions and derivatives formed during processing. The contents of these AAs increased significantly in the Flavourzyme hydrolysates.

Notably, the Papain and Bromelain hydrolysates had significantly lower contents of umami AAs than did the control, whereas the contents of other taste AAs and the total FAA contents were equivalent to those of the control. A previous study also revealed that Papain failed to increase the FAA content in *L. edodes* hydrolysates [10]. This is consistent with their exclusive endopeptidase activity. Due to the absence of corresponding exopeptidase activity, the peptide fragments produced by these enzymes have intact, typically hydrophobic termini that are not further cleaved into FAAs [17]. This specific characteristic also explains the elevated bitterness scores observed in the double-enzyme systems containing these enzymes. Pro and Lys produce a sweet taste at subthreshold levels and a strong bitter taste otherwise. Lys was not detected in any of the samples, and only small amounts of Pro (0.11–0.17 mg/g) were detected in the Flavourzyme, Neutrase, and double-enzyme groups. Notably, all the samples had extremely low quantities of sulfur-containing AAs (Cys and Met), which is consistent with the observations of Gao et al. [16] in morel mushroom hydrolysates.

#### 3.3.3. Equivalent Umami Concentration (EUC) Analysis

EUC has been used to understand the synergistic effect of flavor nucleotides and umami AAs on the overall umami taste [20]. The EUC values were classified into four levels: (1) >1000 g MSG/100 g, (2) 100–1000 g MSG/100 g, (3) 10–100 g MSG/100 g, and (4) <10 g MSG/100 g [32]. The EUC values of the control and Cellulase groups were at level 3, whereas the EUC values of all the other LSHs were at level 2 (Table 2). These results indicate that protease hydrolysis resulted in the production of umami compounds and an enhancement in umami characteristics. The highest EUC value of 288.34 g MSG/100 g was recorded for the Flavourzyme group. These results confirmed that Flavourzyme can release umami substances from *L. edodes* stipes more effectively than other single enzymes or enzyme combinations.

#### 3.3.4. Taste Active Value (TAV) Analysis

The TAV has been used to assess the contribution of non-volatile flavor components to hydrolysate taste [1]. The compounds with TAVs > 1 were considered taste-active, and the higher the value, the greater the contribution of the substance to the taste [1]. As shown in Table 3, Flavourzyme had the greatest number of substances with a TAV > 1 (18), whereas there were 16 substances with a TAV > 1 in the Neutrase, FeN, FeP, and FeB groups. The Cellulase group had the fewest substances with a TAV > 1 (9). Among these taste compounds, Glu had the highest TAV (11.08–19.70), followed by Val (2.81–12.40) and 5′-GMP (2.60–11.96), suggesting that these compounds were the major contributors to LSH taste. Du et al. [1] also reported high TAVs for 5′-GMP and Glu in shiitake mushroom extracts. The TAVs of all four sweet AAs in the Flavourzyme hydrolysates were greater than 1 and significantly higher than the other groups. These sweet AAs mask the bitter taste and synergize with umami AAs to enhance flavor [33]. Interestingly, the TAVs of the bitter AAs in the Flavourzyme, Neutrase, and double-enzyme groups were all greater than 1. However, umami and sweet AAs can easily mask bitter substances [16]. The TAVs of 5′-GMP, 5′-AMP, and 5′-IMP were also greater than 1 in the Flavourzyme and Neutrase hydrolysates and might be responsible for the strong umami taste of these samples.

#### 3.3.5. Sensory Analysis

**Figure 1:**
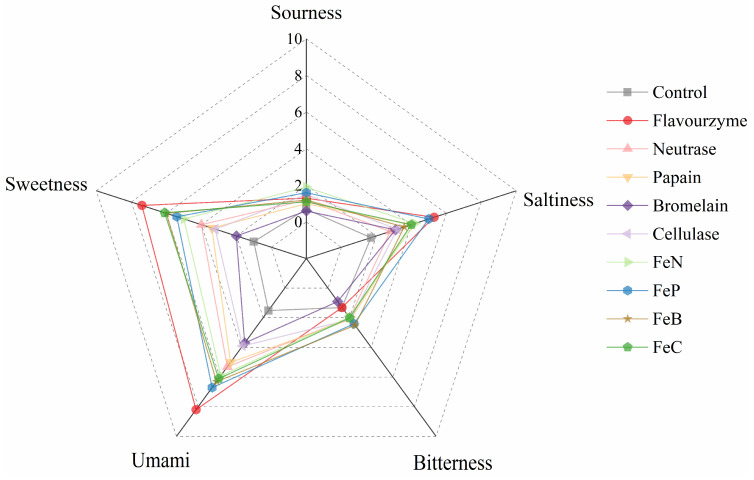
Sensory evaluation of the *Lentinus edodes* stipe hydrolysates (LSHs) and control group.

The control group had the weakest umami, saltiness, and sweetness characteristics, and all of these tastes were enhanced to various degrees after enzymatic hydrolysis (Figure 1). Among the enzyme systems, the Flavourzyme hydrolysates scored the highest for umami, saltiness, and sweetness, followed by the double-enzyme groups. The sensory evaluation results were generally consistent with the DH and FAA results. Saltiness can mask bitterness and increase palatability, and the coexistence of salt and umami can result in an increase in the intensity of each of these tastes [16]. Sweetness is also considered to play an essential and synergistic role with umami in enhancing overall taste [3]. The observation that FeN and FeP in the double-enzyme system resulted in higher bitterness than the Flavourzyme treatment alone likely stems from competitive hydrolysis kinetics and suboptimal reaction conditions. In these systems, the partner endoprotease generated bitter peptides faster than the coupled Flavourzyme exopeptidases could degrade them. This kinetic mismatch led to the net accumulation of bitter compounds. Furthermore, the compromise in optimal conditions such as pH and temperature for the co-hydrolysis might have suppressed the individual activity of each enzyme. This demonstrates that simply combining enzymes does not guarantee synergistic effects. The superior umami and low bitterness scores of the Flavourzyme hydrolysate can be attributed to the extensive cleavage of mushroom proteins into small peptides and free amino acids. Yan et al. [34] demonstrated that low-molecular-weight peptides derived from pea protein exhibited the strongest umami- and saltiness-enhancing effects, highlighting the importance of deep hydrolysis for generating taste-active small molecules. This principle aligns with the present finding that Flavourzyme, with its dual endo- and exopeptidase activities, achieved the highest DH and the most favorable sensory profile among all tested enzyme treatments. These findings indicate that the LSH produced by Flavourzyme has great potential for replacing MSG and NaCl in the production of natural-flavored food products with a strong umami taste and low salt content.

### 3.4. Analysis of the Volatile Compounds in the Lentinus edodes Stipe Hydrolysates (LSHs)

#### 3.4.1. Volatile Compound Profiles

**Figure 2:**
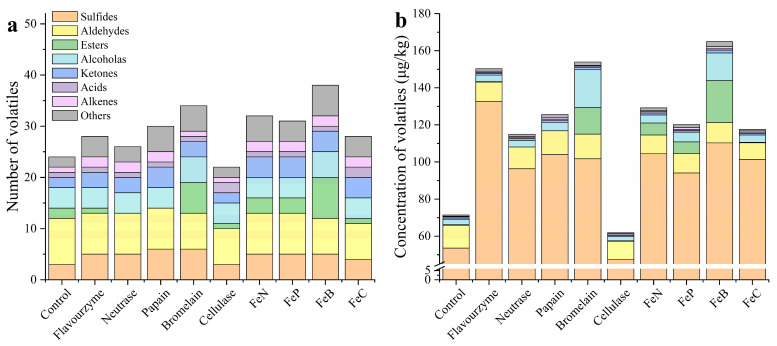
The numbers (**a**) and concentrations (**b**) of the volatile compounds in the *Lentinus edodes* stipe hydrolysates (LSHs) and control group. Statistical comparisons of the total volatile concentrations and individual compound levels are provided in Table S2.

A total of 43 volatile compounds were identified in the LSHs and the control group (Table S2). The differences between the numbers and contents of volatile compounds in the control and LSHs are shown in Figure 2a,b. A total of 24 volatile compounds were detected in the control group, and 19 new volatile compounds were identified in the LSHs, including 7 esters, 2 ketones, 3 sulfides, 1 alcohol, 1 acid, 1 alkene, and 4 others. The numbers and contents of volatile compounds increased to varying degrees in all hydrolysis groups except the Cellulase group. The differences in volatile compounds among the different hydrolysis groups might be correlated with the different protein cleavage sites, as well as the DH [27]. Sulfides are the major volatile compounds in *L. edodes* stipes and account for approximately 75% of the total volatile compound concentration. Notably, the concentration of sulfides in the LSHs increased approximately twofold after protease treatment, significantly exceeding that of the control and Cellulase groups (*p* < 0.05), with Flavourzyme hydrolysates showing the greatest increase (Figure 2b).

The newly created esters, including ethyl butyrate (apple), isoamyl acetate (banana), allyl hexanoate (sweet, pineapple), benzyl acetate (fresh, boiled vegetable), and benzyl propionate (flower, sweet), were present only in the Bromelain and FeB groups, whereas butyl benzoate (balsamic, fruity) and tris(2-chloropropyl) phosphate were present in the double protease-treated LSHs. These esters contribute to the fresh, fruity, and sweet aroma of the LSHs. In addition, minor amounts of 2,3,5-trithiahexane (strong sulfurous onion), 1,2,4,6-tetrathiepane (mushroom-like), and benzyl isothiocyanate were detected after protease hydrolysis, among which benzyl isothiocyanate was detected in the Papain and Bromelain groups only. Newly generated compounds, including 2-heptanone (fruity, sweet, coconut, cheesy), 2-undecanone (fresh, green, orange, rose), (-)-α-cedrene (woody), 1-acetylimidazole, m-toluidine (aromatic aniline-like), and caprolactam (amine spicy), also contributed to the LSH aroma.

#### 3.4.2. Differences in the Volatile Components Among the *Lentinus edodes* Stipe Hydrolysates (LSHs)

**Figure 3:**
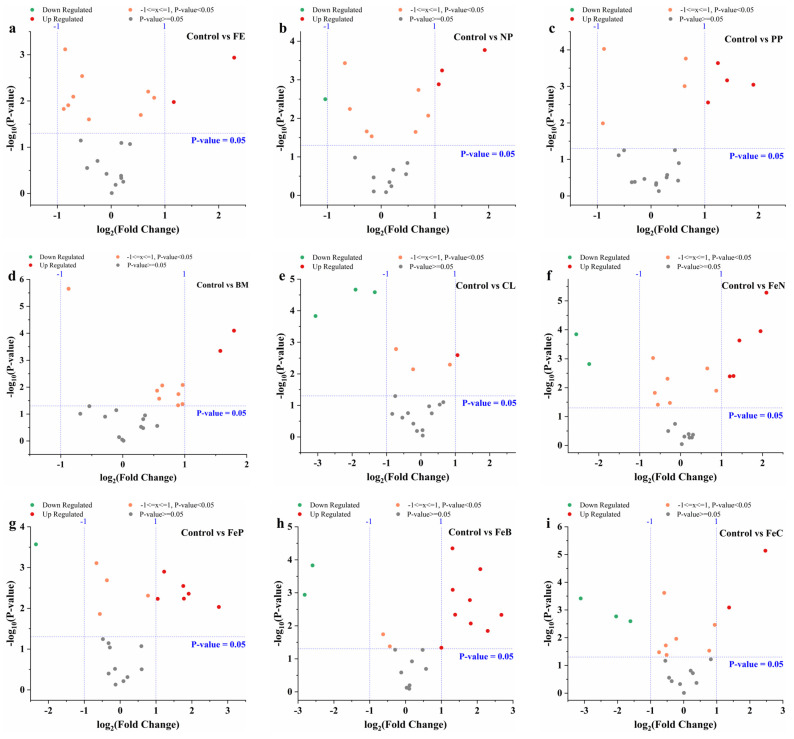
Volcano plot analysis of the volatiles in the *Lentinus edodes* stipe hydrolysates (LSHs) and control group. (**a**) Control vs. Flavourzyme (FE). (**b**) Control vs. Neutrase (NP). (**c**) Control vs. Papain (PP). (**d**) Control vs. Bromelain (BM). (**e**) Control vs. Cellulase (CL). (**f**) Control vs. Flavourzyme-Neutrase (FeN). (**g**) Control vs. Flavourzyme-Papain (FeP). (**h**) Control vs. Flavourzyme-Bromelain (FeB). (**i**) Control vs. Flavourzyme-Cellulase (FeC). In each panel, red dots indicate up-regulated volatiles with *p* < 0.05 and FC > 2; green dots indicate down-regulated volatiles with *p* < 0.05 and FC < 0.5; orange dots indicate volatiles with 0.5 < FC < 2; grey dots indicate volatiles with *p* > 0.05.

Volcano plots were used to analyze the differences in volatile compounds between the control group and LSHs prepared by the different enzyme systems. According to Zhang et al. [27], volatile compounds were considered significantly up-regulated when *p* < 0.05 and fold change (FC) > 2, and significantly down-regulated when *p* < 0.05 and FC < 0.5 (Figure 3a–i). In contrast to the control group, the Flavourzyme, Neutrase, Papain, Bromelain, Cellulase, FeN, FeP, FeB, and FeC groups contained 2, 3, 4, 2, 1, 5, 6, 9, and 2 up-regulated volatile compounds, respectively, which were classified as 3 alcohols, 3 aldehydes, 1 sulfide, 1 alkene, 1 ester, and 1 other compound. In contrast, the Neutrase, Cellulase, FeN, FeP, FeB, and FeC groups contained 1, 3, 2, 1, 2, and 3 down-regulated volatile compounds, respectively, which were classified as 4 aldehydes, 2 sulfides, and 1 other compound.

Cyclic polysulfides are considered the major components that shape the flavor profile of *L. edodes*, imparting meaty, mushroom, onion, charred, and deep-fried flavors [35,36]. The main sulfides identified in the control group were lenthionine, 1,2,4-trithiolane, and 1,2,4,5-tetrathiane. 1,2,4-Trithiolane was significantly up-regulated in all the groups except the Cellulase group. Specifically, the increase in the 1,2,4-trithiolane content was more than 4.5-fold in the Flavourzyme hydrolysates, reaching 103.41 μg/kg. Sulfides are likely produced by the metabolism of sulfur-containing AAs such as cysteine and its derivatives [5,37,38], which was corroborated by the low content of sulfur-containing AAs. 1,2,4-Trithiolane has both sulfuric and oniony odors, and the intensities of these odors are relatively high in *L. edodes* [39]. 1,2,4-Trithiolane was also the most abundant compound and was essential for enhancing LSH aroma. The significant increase in 1,2,4-trithiolane after proteolysis supports the mechanism of precursor release driven by protein hydrolysis. The cyclic polysulfides in *L. edodes* primarily originate from *γ*-glutamylcysteine sulfoxide conjugates and related non-protein sulfur compounds bound within the fungal tissue matrix [35,36]. Protein hydrolysis releases these bound precursors, which are thermally converted to volatile cyclic sulfide.

Alcohols are usually derived from aldehyde reduction or fatty acid degradation [40]. Alcohols not only provide aroma but also serve as solvents for other flavor substances [41]. Significant up-regulation of 1-octen-3-ol was observed in the Flavourzyme, Papain, FeN, FeP, FeB, and FeC groups. Higher levels of this substance were also observed after the enzymatic digestion of oysters by Zhang et al. [27]. 1-Octen-3-ol is derived from linoleic acid breakdown by lipoxygenase and is the main source of mushroom aroma [36,39]. The increase in 1-octene-3-ol content might be related to the type and activity of lipoxygenase and the content and distribution of linoleic acid during enzymatic hydrolysis [27]. Additionally, significant up-regulation of cedrol (cedarwood, sweet, soft) was observed in the Neutrase, Papain, FeN, FeP, and FeB groups, and 2-ethylhexanol (rose, green) was significantly up-regulated in the FeB group. Although cedrol was present at lower levels, it has a low odor threshold and therefore affected the aromas of the LSHs.

Aldehydes usually present a lower threshold and contribute greatly to LSH aroma. Benzaldehyde (almond and burnt sugar) was significantly up-regulated in the Bromelain and FeB groups, and 2-phenylpropenal was significantly up-regulated in the FeP and FeB groups. The increase in the branched aldehyde content might be related to Strecker degradation of AAs and lipid oxidation [27]. However, the significant up-regulation of linear aldehydes such as nonanal (fat, citrus, and green) might be related to the oxidative degradation of unsaturated fatty acids [39,42]. Finally, 2,2,4-trimethyl-1,3-pentanediol diisobutyrate in the double-enzyme groups; α-curcumene (herb) in the Neutrase, Papain, FeN, FeP, and FeB groups; and 2,4-di-tert-butylphenol in the FeB group also increased and promoted LSH aroma.

**Figure 4:**
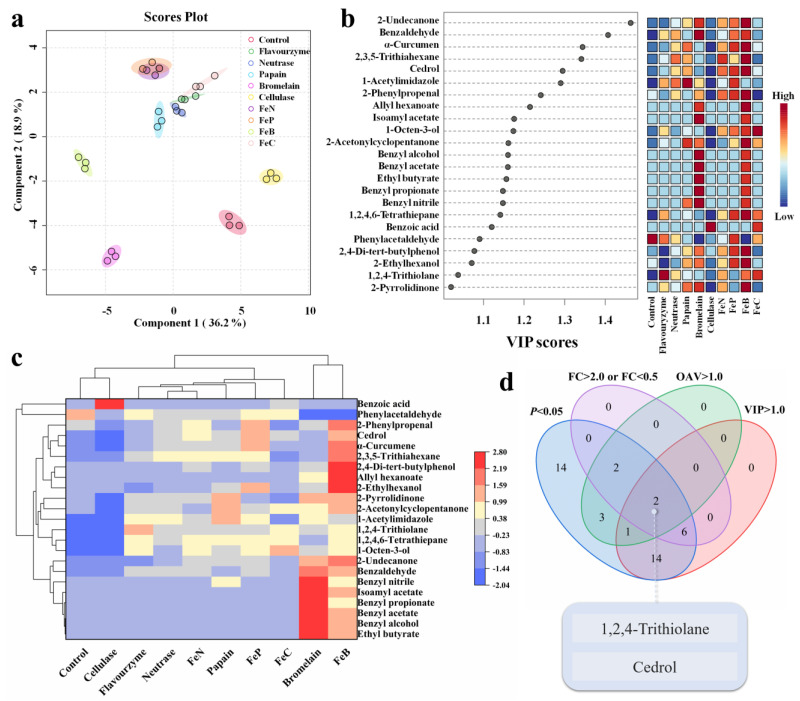
Analysis of the differential volatile compounds in the *Lentinus edodes* stipe hydrolysates (LSHs) and control group. (**a**) PLS-DA score plot. (**b**) volatile compounds with VIP scores > 1. (**c**) Clustering heatmap of the volatile compounds with VIP scores > 1 (*p* < 0.05). (**d**) Venn diagram and key differential aroma compounds.

The volcano plots revealed that the numbers of up- and down-regulation of volatile compounds in the LSHs varied, and the optimal protease could not be identified by these results alone. Hence, differential analysis of the volatile compounds in the LSHs was carried out via PLS-DA. The PLS-DA score plot revealed that PC1 and PC2 explained 36.2% and 18.9% of the total variance, respectively (Figure 4a). Interestingly, the control and Cellulase groups were located in quadrant IV, whereas the Bromelain and FeB groups were located in quadrant III, while the other groups were closer to each other. The volatile compound compositions of the LSHs in the same region were relatively similar, which proved that protease hydrolysis altered the flavor profile of *L. edodes* stipes. The variable importance profile (VIP) scores of the volatiles in the samples were derived via PLS-DA. A VIP > 1 (*p* < 0.05) was employed to identify important volatile compounds in the different LSHs [43]. A total of 23 volatile compounds had a VIP value of >1 (*p* < 0.05), as shown in Figure 4b.

A clustering heatmap was used to visualize the differences in compounds with VIP > 1 among the different LSHs. As shown in Figure 4c, the LSHs were classified into three clusters: the first cluster included the control and Cellulase groups; the second cluster included the Flavourzyme, Neutrase, Papain, FeC, FeN, and FeP groups; and the Bromelain and FeB groups were placed in the third cluster. These findings indicated that the flavor of the protease-hydrolyzed LSH differed significantly from those of the control and Cellulase hydrolysates and that protease hydrolysis significantly increased the production of volatiles in the LSHs. According to cluster analysis, FeB treatment presented the greatest number of volatile compounds with fruity and floral aromas, followed by Bromelain.

LSH flavor is dependent on both the odor threshold and the composition and concentration of volatile compounds. Volatile compounds with OAVs ≥ 1 were considered the main agents influencing sample flavor [27]. There were 9 compounds with OAVs ≥ 1, including 2 sulfides, 4 aldehydes, 2 esters, and 1 alcohol (Table S3). 1,2,4,5-Tetrathiane (OAV: 10.37–86.66) and 1,2,4-trithiolane (OAV: 2.45–13.50) were the main contributors to the mushroom aroma of *L. edodes* stipes. The OAV of 1,2,4,5-tetrathiane decreased after enzyme hydrolysis, whereas the OAV of 1,2,4-trithiolane increased significantly. The decrease in the OAV of 1,2,4,5-tetrathiane might be related to its volatility [35]. Hexanal (OAV: 0.82–1.44), nonanal (OAV: 1.01–2.32), (E)-2-nonenal (OAV: 1.05–3.70), and 2,4-dimethylbenzaldehyde (OAV: 0.85–9.62) impart green, fatty, and fruity aromas to the LSHs. The OAVs of 2,4-dimethylbenzaldehyde in the single-enzyme hydrolysates were all > 5, lower in the double-enzyme hydrolysates, and even below the threshold in the FeN and FeB groups. Ethyl butyrate (OAV: 5.04–9.96), which has a fruity and sweet aroma, appeared only in the Bromelain and FeB groups. 2,2,4-Trimethyl-1,3-pentanediol diisobutyrate (OAV: 1.00–6.38) had higher OAVs in the double protease groups than in the other groups. The OAVs of cedrol were below the threshold in the control and Cellulase groups but significantly increased upon protease hydrolysis (OAV: 1.46–3.31). Volatile compounds with FC > 2 or FC < 0.5, VIP > 1, *p* < 0.05, and OAV > 1 were considered key aroma compounds [27]. In summary, 1,2,4-trithiolane and cedrol fulfilled these conditions and were identified as the key compounds responsible for the different aromas of the LSHs (Figure 4d).

## 4. Conclusions

This study demonstrated that Flavourzyme alone outperforms all tested enzymatic combinations for producing high-umami and low-bitterness hydrolysates from *L. edodes* stipes. The Flavourzyme hydrolysate exhibited the highest degree of hydrolysis and the most favorable sensory profile, driven by abundant umami amino acids and 5′-nucleotides. Notably, all double-enzyme combinations underperformed Flavourzyme alone, indicating that indiscriminate co-addition of commercial enzymes does not guarantee synergistic flavor enhancement and may be detrimental. Multivariate analysis identified 1,2,4-trithiolane and cedrol as the key volatile markers distinguishing the hydrolysates. These findings establish Flavourzyme as the preferred enzyme for converting *L. edodes* stipes into clean-tasting, umami-rich condiments. Future research should explore sequential hydrolysis strategies to further improve protein recovery rates, characterise the umami peptides responsible for taste enhancement, and quantify the contribution of non-protein components (such as soluble carbohydrates and minerals) to the overall flavor profile, to provide a comprehensive understanding of the role of enzymatic hydrolysis in enhancing the flavor and umami of *L. edodes* stipes.

## Figures and Tables

**Table 1:** Protein recovery (PR), degree of hydrolysis (DH), and color values of the *Lentinus edodes* stipe hydrolysates (LSHs) and control group.

	PR (%)	DH (%)	Color			
			L*	a*	b*	ΔE
Control	40.51 ± 2.39 ^g^	19.16 ± 1.20 ^e^	43.56 ± 0.30 ^b^	14.18 ± 0.12 ^e^	36.38 ± 0.38 ^b^	-
Flavourzyme	47.56 ± 0.48 ^f^	50.38 ± 1.68 ^a^	33.88 ± 0.07 ^d^	16.16 ± 0.15 ^abc^	24.03 ± 0.16 ^d^	15.82 ± 0.15 ^bc^
Neutrase	47.92 ± 0.21 ^f^	39.91 ± 1.57 ^b^	33.28 ± 0.17 ^de^	15.59 ± 0.39 ^cd^	22.31 ± 0.43 ^ef^	17.49 ± 0.41 ^ab^
Papain	56.65 ± 1.80 ^b^	24.66 ± 2.94 ^c^	32.92 ± 0.42 ^f^	15.97 ± 0.48 ^bcd^	21.82 ± 0.89 ^f^	18.13 ± 0.91 ^a^
Bromelain	54.34 ± 0.36 ^bc^	23.39 ± 3.21 ^cd^	35.44 ± 0.16 ^c^	16.67 ± 0.07 ^a^	26.13 ± 0.28 ^c^	13.32 ± 0.31 ^d^
Cellulase	62.90 ± 0.58 ^a^	20.77 ± 1.46 ^de^	48.63 ± 0.34 ^a^	11.77 ± 0.14 ^f^	42.34 ± 0.54 ^a^	8.19 ± 0.56 ^e^
FeN	49.19 ± 0.11 ^ef^	47.52 ± 2.54 ^a^	33.81 ± 0.34 ^d^	16.08 ± 0.07 ^abc^	23.81 ± 0.59 ^de^	16.03 ± 0.66 ^b^
FeP	50.73 ± 2.05 ^de^	48.24 ± 1.52 ^a^	33.08 ± 0.69 ^de^	15.80 ± 0.62 ^bcd^	23.86 ± 1.57 ^de^	16.48 ± 1.08 ^b^
FeB	52.53 ± 1.72 ^cd^	46.90 ± 1.84 ^a^	32.78 ± 1.09 ^f^	15.43 ± 0.55 ^d^	21.76 ± 1.82 ^f^	18.22 ± 2.08 ^a^
FeC	60.64 ± 1.61 ^a^	41.49 ± 1.56 ^b^	34.80 ± 0.04 ^c^	16.32 ± 0.20 ^ab^	25.19 ± 0.10 ^cd^	14.38 ± 0.07 ^cd^

Different lowercase letters in the same column indicate significant differences (*p* < 0.05). FeN: Flavourzyme-Neutrase; FeP: Flavourzyme-Papain; FeB: Flavourzyme-Bromelain; FeC: Flavourzyme-Cellulase.

**Table 2:** Free amino acids (FAAs) and 5′-nucleotide contents and equivalent umami concentration (EUC) values in the *Lentinus edodes* stipe hydrolysates (LSHs) and control group.

	Control	Flavourzyme	Neutrase	Papain	Bromelain	Cellulase	FeN	FeP	FeB	FeC
**FAAs (mg/g db)**										
**Umami**										
Asp	1.35 ± 0.10 ^e^	2.32 ± 0.19 ^a^	1.33 ± 0.07 ^e^	1.22 ± 0.15 ^ef^	1.18 ± 0.00 ^ef^	0.92 ± 0.01 ^f^	1.70 ± 0.15 ^cd^	2.04 ± 0.07 ^b^	1.84 ± 0.35 ^bc^	1.44 ± 0.22 ^de^
Glu	4.71 ± 0.06 ^c^	5.91 ± 0.25 ^a^	3.88 ± 0.13 ^e^	3.55 ± 0.05 ^f^	4.31 ± 0.13 ^d^	3.32 ± 0.08 ^f^	5.03 ± 0.32 ^b^	4.83 ± 0.06 ^bc^	4.89 ± 0.11 ^bc^	4.88 ± 0.11 ^bc^
Total	6.05 ± 0.09 ^d^	8.24 ± 0.22 ^a^	5.22 ± 0.16 ^e^	4.77 ± 0.19 ^f^	5.49 ± 0.13 ^e^	4.24 ± 0.09 ^g^	6.73 ± 0.47 ^c^	6.87 ± 0.08 ^b^	6.72 ± 0.43 ^bc^	6.32 ± 0.33 ^cd^
**Sweet**										
Thr	2.24 ± 0.06 ^e^	4.95 ± 0.13 ^a^	3.19 ± 0.09 ^d^	2.31± 0.19 ^e^	2.42 ± 0.04 ^e^	1.31 ± 0.02 ^f^	3.88 ± 0.30 ^b^	3.71 ± 0.20 ^b^	3.64 ± 0.10 ^bc^	3.43 ± 0.20 ^cd^
Ser	1.11 ± 0.04 ^e^	3.28 ± 0.15 ^a^	1.53 ± 0.04 ^d^	1.23 ± 0.12 ^e^	1.29 ± 0.03 ^de^	0.75 ± 0.01 ^f^	2.61 ± 0.28 ^b^	2.70 ± 0.25 ^b^	2.50 ± 0.08 ^b^	2.118 ± 0.27 ^c^
Gly	0.55 ± 0.01 ^e^	1.41 ± 0.02 ^a^	0.68 ± 0.03 ^d^	0.55 ± 0.02 ^e^	0.59 ± 0.02 ^de^	0.39 ± 0.01 ^f^	1.12 ± 0.15 ^b^	1.15 ± 0.11 ^b^	1.07 ± 0.03 ^b^	0.85 ± 0.07 ^c^
Ala	3.74 ± 0.11 ^e^	5.57 ± 0.06 ^a^	4.34 ± 0.14 ^cd^	3.67 ± 0.20 ^e^	3.81 ± 0.06 ^e^	2.61 ± 0.03 ^f^	4.70 ± 0.31 ^b^	4.51 ± 0.12 ^bc^	4.51 ± 0.06 ^bc^	4.16 ± 0.20 ^d^
Total	7.64 ± 0.17 ^d^	15.21 ± 0.31 ^a^	9.73 ± 0.27 ^c^	7.77 ± 0.53 ^d^	8.12 ± 0.16 ^d^	5.07 ± 0.07 ^e^	12.31 ± 1.02 ^b^	12.06 ± 0.69 ^b^	11.72 ± 0.26 ^b^	10.55 ± 0.73 ^c^
**Bitter**										
Val	2.02 ± 0.06 ^f^	4.96 ± 0.07 ^a^	4.57 ± 0.12 ^bc^	2.05 ± 0.20 ^f^	2.09 ± 0.08 ^f^	1.12 ± 0.02 ^g^	4.19 ± 0.28 ^d^	4.34 ± 0.41 ^cd^	4.67 ± 0.09 ^ab^	3.63 ± 0.13 ^e^
Met	0.25 ± 0.00 ^f^	0.74 ± 0.03 ^a^	0.53 ± 0.01 ^e^	0.27 ± 0.02 ^f^	0.21 ± 0.01 ^fg^	0.18 ± 0.01 ^g^	0.59 ± 0.07 ^de^	0.65 ± 0.06 ^bc^	0.69 ± 0.01 ^ab^	0.61 ± 0.02 ^cd^
Ile	1.73 ± 0.03 ^f^	5.48 ± 0.06 ^a^	5.20 ± 0.13 ^b^	1.80 ± 0.27 ^f^	1.71 ± 0.17 ^f^	1.02 ± 0.03 ^g^	4.69 ± 0.28 ^c^	4.40 ± 0.13 ^de^	4.52 ± 0.08 ^cd^	4.18 ± 0.11 ^e^
Leu	2.39 ± 0.02 ^d^	8.05 ± 0.10 ^a^	6.91 ± 0.22 ^b^	2.70 ± 0.38 ^d^	2.66 ± 0.25 ^d^	1.61 ± 0.06 ^e^	6.90 ± 0.40 ^b^	6.54 ± 0.08 ^bc^	6.76 ± 0.10 ^b^	6.16 ± 0.20 ^c^
Phe	1.90 ± 0.03 ^e^	6.06 ± 0.10 ^a^	4.96 ± 0.15 ^bc^	1.94 ± 0.18 ^e^	2.08 ± 0.11 ^e^	1.48 ± 0.16 ^f^	5.03 ± 0.32 ^b^	4.62 ± 0.10 ^d^	4.72 ± 0.06 ^cd^	4.67 ± 0.12 ^d^
His	0.26 ± 0.00 ^d^	1.18 ± 0.09 ^a^	0.42 ± 0.03 ^c^	0.21 ± 0.02 ^d^	0.26 ± 0.01 ^d^	0.18 ± 0.00 ^d^	0.85 ± 0.07 ^b^	0.89 ± 0.06 ^b^	0.83 ± 0.06 ^b^	0.83 ± 0.06 ^b^
Arg	1.70 ± 0.06 ^f^	4.54 ± 0.13 ^a^	2.83 ± 0.13 ^d^	1.72 ± 0.15 ^f^	1.96 ± 0.12 ^e^	1.38 ± 0.05 ^g^	3.69 ± 0.21 ^b^	3.68 ± 0.07 ^b^	3.76 ± 0.07 ^b^	3.33 ± 0.18 ^c^
Total	10.26 ± 0.01 ^d^	31.00 ± 0.39 ^a^	25.42 ± 0.77 ^b^	10.69 ± 1.22 ^d^	10.97 ± 0.75 ^d^	6.98 ± 0.31 ^e^	25.95 ± 1.61 ^b^	25.12 ± 0.56 ^b^	25.93 ± 0.45 ^b^	23.41 ± 0.82 ^c^
**Sweet/Bitter**										
Lys	ND	ND	ND	ND	ND	ND	ND	ND	ND	ND
Pro	ND	0.17 ± 0.00 ^a^	0.13 ± 0.01 ^b^	ND	ND	ND	0.14 ± 0.02 ^b^	0.12 ± 0.00 ^b^	0.11 ± 0.01 ^b^	0.13 ± 0.02 ^b^
Total	ND	0.17 ± 0.00 ^a^	0.13 ± 0.01 ^b^	ND	ND	ND	0.14 ± 0.02 ^b^	0.12 ± 0.00 ^b^	0.11 ± 0.01 ^b^	0.13 ± 0.02 ^b^
**Tasteless**										
Cys	0.60 ± 0.06 ^c^	0.77 ± 0.08 ^a^	0.73 ± 0.01 ^ab^	0.50 ± 0.03 ^d^	0.46 ± 0.00 ^d^	0.42 ± 0.00 ^d^	0.68 ± 0.07 ^abc^	0.69 ± 0.11 ^abc^	0.76 ± 0.02 ^a^	0.66 ± 0.01 ^bc^
Tyr	0.70 ± 0.01 ^f^	3.68 ± 0.04 ^a^	2.29 ± 0.07 ^d^	0.95 ± 0.08 ^e^	1.00 ± 0.05 ^e^	0.56 ± 0.14 ^f^	2.89 ± 0.29 ^b^	2.79 ± 0.10 ^b^	2.78 ± 0.05 ^b^	2.56 ± 0.13 ^c^
Total	1.30 ± 0.07 ^d^	4.44 ± 0.12 ^a^	3.01 ± 0.08 ^c^	1.45 ± 0.10 ^d^	1.47 ± 0.05 ^d^	0.98 ± 0.15 ^e^	3.57 ± 0.36 ^b^	3.48 ± 0.04 ^b^	3.54 ± 0.05 ^b^	3.22 ± 0.14 ^c^
EAAs	10.80 ± 0.11 ^d^	31.41 ± 0.38 ^a^	25.78 ± 0.71 ^b^	11.28 ± 1.26 ^d^	11.43 ± 0.67 ^d^	6.92 ± 0.27 ^e^	26.15 ± 1.68 ^b^	25.15 ± 0.40 ^b^	25.82 ± 0.48 ^b^	23.52 ± 0.84 ^c^
Grand total	25.25 ± 0.30 ^d^	59.06 ± 0.47 ^a^	43.51 ± 1.19 ^c^	24.68 ± 2.04 ^d^	26.04 ± 0.94 ^d^	17.28 ± 0.57 ^e^	48.69 ± 3.45 ^b^	47.65 ± 0.61 ^b^	48.03 ± 1.07 ^b^	43.64 ± 2.02 ^c^
**5′-Nucleotides (mg/g db)**										
5′-CMP	1.13 ± 0.09 ^a^	1.16 ± 0.10 ^a^	1.17 ±0.05 ^a^	0.89 ± 0.13 ^b^	0.86 ± 0.09 ^b^	0.56 ± 0.07 ^c^	0.58 ± 0.06 ^c^	0.57 ± 0.11 ^c^	1.10 ± 0.05 ^a^	0.67 ± 0.14 ^c^
5′-AMP	0.20 ± 0.01 ^d^	0.54 ± 0.06 ^a^	0.53 ± 0.04 ^a^	0.40 ± 0.02 ^b^	0.31 ± 0.08 ^c^	0.02 ± 0.00 ^e^	0.19 ± 0.01 ^d^	0.17 ± 0.00 ^d^	0.07 ± 0.02 ^e^	0.16 ± 0.03 ^d^
5′-GMP	0.46 ± 0.01 ^de^	1.46 ± 0.09 ^a^	1.50 ± 0.11 ^a^	1.36 ± 0.07 ^a^	1.08 ± 0.17 ^b^	0.32 ± 0.03 ^e^	0.67 ± 0.05 ^c^	0.69 ± 0.11 ^c^	0.59 ± 0.10 ^cd^	0.71 ± 0.07 ^c^
5′-UMP	0.74 ± 0.06 ^e^	1.87 ± 0.03 ^a^	1.89 ± 0.02 ^a^	1.64 ± 0.08 ^b^	1.44 ± 0.13 ^c^	0.30 ± 0.03 ^f^	1.13 ± 0.04 ^d^	1.08 ± 0.06 ^d^	0.81 ± 0.02 ^e^	1.20 ± 0.11 ^d^
5′-IMP	0.30 ± 0.05 ^d^	0.42 ± 0.04 ^c^	0.42 ± 0.04 ^c^	0.62 ± 0.05 ^a^	0.63 ± 0.04 ^a^	0.25 ± 0.01 ^d^	0.54 ± 0.05 ^ab^	0.57 ± 0.01 ^ab^	0.50 ± 0.06 ^bc^	0.62 ± 0.04 ^a^
Total	2.83 ± 0.21 ^d^	5.46 ± 0.10 ^a^	5.50 ± 0.25 ^a^	4.91 ± 0.25 ^b^	4.33 ± 0.45 ^c^	1.44 ± 0.06 ^e^	3.11 ± 0.20 ^d^	3.08 ± 0.53 ^d^	3.08 ± 0.13 ^d^	3.36 ± 0.29 ^d^
**EUC (g MSG/100 g db)**	82.42 ± 3.24 ^d^	288.34 ± 8.44 ^a^	192.16 ± 10.68 ^b^	170.41 ± 11.97 ^b^	170.31 ± 18.71 ^b^	41.71 ± 2.24 ^e^	132.48 ± 2.01 ^c^	133.75 ± 26.17 ^c^	115.66 ± 17.05 ^c^	139.78 ± 11.16 ^c^

Different lowercase letters in the same row indicate significant differences (*p* < 0.05). EAAs: essential amino acids including Thr, Val, Met, Ile, Leu, Phe, His and Lys. Tryptophan was not determined. ND: not detected.

**Table 3:** Taste active values (TAVs) of the *Lentinus edodes* stipe hydrolysates (LSHs) and control group.

	Threshold Value (mg/g)	TAVs									
		Control	Flavourzyme	Neutrase	Papain	Bromelain	Cellulase	FeN	FeP	FeB	FeC
**FAAs**											
Asp	1	1.35 ± 0.10	2.32 ± 0.19	1.33 ± 0.07	1.22 ± 0.15	1.18 ± 0.00	0.92 ± 0.01	1.70 ± 0.15	2.04 ± 0.07	1.84 ± 0.35	1.44 ± 0.22
Glu	0.3	15.68 ± 0.19	19.70 ± 0.82	12.95 ± 0.43	11.83 ± 0.16	14.38 ± 0.43	11.08 ± 0.27	16.76 ± 1.07	16.10 ± 0.20	16.30 ± 0.36	16.28 ± 0.36
Thr	2.6	0.86 ± 0.02	1.90 ± 0.05	1.23 ± 0.03	0.89 ± 0.07	0.93 ± 0.02	0.51 ± 0.01	1.49 ± 0.12	1.43 ± 0.08	1.40 ± 0.04	1.32 ± 0.08
Ser	1.5	0.74 ± 0.03	2.19 ± 0.10	1.02 ± 0.03	0.82 ± 0.08	0.86 ± 0.02	0.50 ± 0.01	1.74 ± 0.18	1.80 ± 0.17	1.67 ± 0.05	1.41 ± 0.18
Gly	1.3	0.42 ± 0.01	1.08 ± 0.01	0.52 ± 0.02	0.43 ± 0.02	0.45 ± 0.01	0.30 ± 0.00	0.86 ± 0.11	0.88 ± 0.08	0.82 ± 0.02	0.65 ± 0.06
Ala	0.6	6.23 ± 0.18	9.29 ± 0.10	7.23 ± 0.24	6.12 ± 0.33	6.35 ± 0.11	4.35 ± 0.06	7.83 ± 0.52	7.51 ± 0.21	7.52 ± 0.10	6.93 ± 0.33
Val	0.4	5.06 ± 0.14	12.40 ± 0.17	11.42 ± 0.30	5.12 ± 0.51	5.21 ± 0.20	2.81 ± 0.04	10.49 ± 0.70	10.85 ± 1.02	11.67 ± 0.21	9.08 ± 0.33
Met	0.3	0.83 ± 0.01	2.46 ± 0.10	1.78 ± 0.04	0.90 ± 0.07	0.70 ± 0.03	0.59 ± 0.03	1.97 ± 0.23	2.18 ± 0.21	2.28 ± 0.04	2.03 ± 0.08
Ile	0.9	1.92 ± 0.04	6.09 ± 0.06	5.78 ± 0.14	2.00 ± 0.30	1.90 ± 0.19	1.13 ± 0.03	5.22 ± 0.31	4.89 ± 0.14	5.02 ± 0.08	4.64 ± 0.13
Leu	1.9	1.26 ± 0.01	4.23 ± 0.05	3.64 ± 0.12	1.42 ± 0.20	1.40 ± 0.13	0.85 ± 0.03	3.63 ± 0.21	3.44 ± 0.04	3.56 ± 0.05	3.24 ± 0.11
Phe	0.9	2.11 ± 0.04	6.74 ± 0.11	5.51 ± 0.17	2.15 ± 0.20	2.31 ± 0.13	1.65 ± 0.18	5.59 ± 0.35	5.13 ± 0.11	5.24 ± 0.07	5.19 ± 0.14
His	0.2	1.30 ± 0.02	5.89 ± 0.46	2.10 ± 0.15	1.06 ± 0.11	1.31 ± 0.06	0.92 ± 0.01	4.25 ± 0.36	4.44 ± 0.29	4.14 ± 0.32	4.15 ± 0.32
Arg	0.5	3.40 ± 0.13	9.08 ± 0.25	5.65 ± 0.26	3.44 ± 0.29	3.92 ± 0.24	2.76 ± 0.10	7.37 ± 0.42	7.36 ± 0.14	7.52 ± 0.14	6.65 ± 0.36
Lys	0.5	ND	ND	ND	ND	ND	ND	ND	ND	ND	ND
Pro	3	ND	0.06 ± 0.00	0.04 ± 0.00	ND	ND	ND	0.05 ± 0.01	0.04 ±0.00	0.04 ± 0.00	0.04 ± 0.01
Cys	0.4	1.50 ± 0.14	1.92 ± 0.20	1.82 ± 0.03	1.24 ± 0.06	1.16 ± 0.01	1.06 ± 0.01	1.70 ± 0.18	1.73 ± 0.26	1.89 ± 0.04	1.64 ± 0.03
Tyr	2.6	0.27 ± 0.00	1.41 ± 0.01	0.88 ± 0.03	0.37 ± 0.03	0.39 ± 0.02	0.21 ± 0.05	1.11 ± 0.11	1.07 ± 0.04	1.07 ± 0.02	0.99 ± 0.05
**5′-Nucleotides**											
5′-CMP	/	/	/	/	/	/	/	/	/	/	/
5′-AMP	0.5	0.40 ± 0.03	1.08 ± 0.11	1.06 ± 0.09	0.80 ± 0.04	0.62 ± 0.17	0.03 ± 0.00	0.38 ± 0.02	0.35 ± 0.09	0.14 ± 0.03	0.31 ± 0.07
5′-GMP	0.125	3.70 ± 0.07	11.71 ± 0.74	11.96 ± 0.88	10.92 ±0.55	8.66 ± 1.35	2.60 ± 0.25	5.34 ± 0.39	5.52 ± 1.09	4.76 ± 0.81	5.69 ± 0.56
5′-UMP	5.5	0.13 ± 0.01	0.34 ± 0.01	0.34 ± 0.00	0.30 ± 0.01	0.26 ± 0.02	0.05 ± 0.00	0.21 ± 0.01	0.20 ± 0.03	0.15 ± 0.00	0.22 ± 0.02
5′-IMP	0.25	1.20 ± 0.22	1.69 ± 0.14	1.68 ± 0.16	2.48 ± 0.22	2.52 ± 0.15	1.01 ± 0.02	2.15 ± 0.20	2.29 ± 0.43	1.98 ± 0.24	2.49 ± 0.15

ND: not detected. “/” means no threshold was found.

## Data Availability

The original contributions presented in this study are included in the article/Supplementary Materials. Further inquiries can be directed to the corresponding author.

## Supplementary Materials

The following supporting information can be downloaded at: https://www.mdpi.com/article/10.3390/foods15142495/s1, Table S1: The main nutrients in *Lentinus edodes* and *Lentinus edodes* stipes; Table S2: Content of volatile compounds in the *Lentinus edodes* stipe hydrolysates (LSHs) and control group; Table S3: Odor threshold and odor activity value (OAV) in the *Lentinus edodes* stipe hydrolysates (LSHs) and control group.
