# Supplementary material for: Analysis of the Umami Taste and Volatile Flavor Components of Lentinus edodes Stipe Hydrolysates Derived After Different Enzymatic Treatments
Source: Foods. 2026 Jul 14;15(14):2495. doi: 10.3390/foods15142495 (PMC13407709; doi:10.3390/foods15142495)
Supplement: Supplementary file 1 [file foods-15-02495-s001.zip › foods-4380456-supplementary materials.pdf]

Table S1: The main nutrients in *Lentinus edodes* and *Lentinus edodes* stipes.

	Moisture content (g/100 g)	Total protein (g/100 g db)	Total sugar (g/100 g db)	Crude fiber (g/100 g db)	Fat (g/100 g db)	Ash (g/100 g db)
L. edodes	9.77 ± 0.60	21.27 ± 0.42	31.64 ± 0.64	22.05 ± 0.15	2.78 ± 0.12	5.22 ± 0.30
L. edodes stipes	8.57 ± 1.07	15.24 ± 0.99	32.30 ± 1.65	26.07 ± 0.12	2.95 ± 0.21	4.77 ± 0.19

db: dry weight basis.

Table S2 Content of volatile compounds in the *Lentinus edodes* stipe hydrolysates (LSHs) and control group.

Compounds	CAS	Formula	Content (μg/kg)									
			Control	Flavourzyme	Neutrase	Papain	Bromelain	Cellulase	FeN	FeP	FeB	FeC
Aldehydes												
Hexanal	66-25-1	$C_6H_{12}O$	6.07 ± 0.45^{ab}	4.56 ± 0.60^{cde}	5.04 ± 0.20^{bcd}	6.49 ± 0.74^{a}	5.83 ± 1.02^{abc}	3.68 ± 0.32^{e}	5.06 ± 0.32^{bcd}	4.97 ± 0.73^{bcd}	5.58 ± 0.46^{abc}	4.14 ± 1.27^{de}
Heptanal	111-71-7	$C_7H_{14}O$	0.22 ± 0.03^{a}	0.13 ± 0.02^{bc}	0.11 ± 0.00^{c}	0.14 ± 0.04^{bc}	0.15 ± 0.03^{bc}	0.26 ± 0.01^{a}	0.14 ± 0.01^{bc}	0.16 ± 0.03^{bc}	0.16 ± 0.01^{b}	0.15 ± 0.01^{bc}
(E)-2-Heptenal	18829-55-5	$C_7H_{12}O$	0.34 ± 0.03^{a}	ND	ND	ND	ND	ND	ND	ND	ND	ND
Benzaldehyde	100-52-7	C_7H_6O	1.04 ± 0.10^{d}	1.68 ± 0.18^{c}	1.69 ± 0.11^{c}	1.61 ± 0.04^{c}	3.10 ± 0.32^{a}	1.08 ± 0.03^{d}	1.63 ± 0.10^{c}	1.78 ± 0.20^{c}	2.58 ± 0.09^{b}	1.36 ± 0.62^{cd}
Phenylacetaldehyde	122-78-1	C_8H_8O	1.75 ± 0.07^{a}	1.20 ± 0.12^{c}	1.10 ± 0.07^{cd}	0.95 ± 0.04^{d}	ND	0.69 ± 0.04^{e}	1.10 ± 0.11^{cd}	1.40 ± 0.24^{b}	ND	1.16 ± 0.03^{c}
Nonanal	124-19-6	$C_9H_{18}O$	1.11 ± 0.09^{bc}	1.41 ± 0.22^{b}	1.30 ± 0.21^{bc}	1.18 ± 0.15^{bc}	1.10 ± 0.01^{c}	2.32 ± 0.30^{a}	1.01 ± 0.06^{c}	1.01 ± 0.13^{c}	1.26 ± 0.10^{bc}	1.29 ± 0.15^{bc}
(E)-2-Nonenal	18829-56-6	$C_9H_{16}O$	0.36 ± 0.02^{b}	0.20 ± 0.02^{cd}	0.56 ± 0.09^{a}	0.56 ± 0.02^{a}	0.70 ± 0.20^{a}	0.10 ± 0.01^{d}	0.29 ± 0.10^{bc}	0.23 ± 0.02^{bcd}	0.29 ± 0.05^{bc}	0.22 ± 0.08^{bcd}
2-Phenylpropenal	4432-63-7	C_9H_8O	0.37 ± 0.06^{de}	0.25 ± 0.06^{e}	0.52 ± 0.13^{cd}	0.30 ± 0.12^{e}	0.54 ± 0.04^{c}	ND	0.67 ± 0.11^{bc}	0.76 ± 0.11^{b}	0.96 ± 0.17^{a}	ND

2,4-Dimethylbenzaldehyde	15764-16-6	$C_9H_{10}O$	1.03±0.10^{cd}	1.18±0.03^{bcd}	1.42±0.53^{abc}	1.48±0.39^{abc}	1.92±0.39^{a}	1.62±0.42^{ab}	0.18±0.01^{e}	0.20±0.06^{e}	0.17±0.02^{e}	0.72±0.15^{d}
**Total**			12.29 ± 0.66^{abc}	10.61 ± 1.06^{cdef}	11.73 ± 0.45^{abcd}	12.72±0.38^{ab}	13.35±1.05^{a}	9.74±0.95^{ef}	10.08±0.60^{def}	10.51±1.13^{cdef}	11.01 ± 0.52^{bcde}	9.03±1.90^{f}
Esters												
Ethyl butyrate	105-54-4	$C_6H_{12}O_2$	ND	ND	ND	ND	9.96±0.76^{a}	ND	ND	ND	5.04±0.65^{b}	ND
Isoamyl acetate	123-92-2	$C_7H_{14}O_2$	ND	ND	ND	ND	1.10±0.12^{a}	ND	ND	ND	0.70±0.06^{b}	ND
Allyl hexanoate	123-68-2	$C_9H_{16}O_2$	ND	ND	ND	ND	2.30±0.29^{b}	ND	ND	ND	6.51±3.53^{a}	ND
Benzyl acetate	140-11-4	$C_9H_{10}O_2$	ND	ND	ND	ND	0.61±0.05^{a}	ND	ND	ND	0.32±0.04^{b}	ND
Benzyl propionate	122-63-4	$C_{10}H_{12}O_2$	ND	ND	ND	ND	0.16±0.01^{a}	ND	ND	ND	0.07±0.01^{b}	ND
Butyl benzoate	136-60-7	C_{11}H14O_2	ND	ND	ND	ND	ND	ND	0.13±0.01^{b}	0.09±0.01^{c}	0.16±0.03^{a}	ND
Tris(2-chloropropyl) phosphate	6145-73-9	$C_9H_{18}Cl_3O_4P$	ND	ND	ND	ND	ND	ND	5.80±0.23^{b}	5.23±0.47^{b}	8.98±3.64^{a}	ND
Triisobutyl phosphate	126-71-6	$C_{12}H_{27}O_4P$	0.19±0.06^{a}	ND	ND	ND	ND	ND	ND	ND	ND	ND
2,2,4-Trimethyl-1,3-pentanediol diisobutyrate	6846-50-0	$C_{16}H_{30}O_4$	0.13±0.01^{cd}	0.15±0.04^{cd}	ND	ND	0.24±0.07^{c}	0.23±0.03^{c}	0.56±0.02^{b}	0.88±0.27^{a}	0.83±0.21^{a}	0.23±0.06^{c}
**Total**			0.32±0.06^{d}	0.15±0.04^{d}	ND	ND	14.36±0.76^{b}	0.23±0.03^{d}	6.49±0.21^{c}	6.19±0.76^{c}	22.62±4.32^{a}	0.23±0.06^{d}
Ketones												
2-Heptanone	110-43-0	$C_7H_{14}O$	ND	0.10±0.01^{d}	ND	0.12±0.02^{bc}	ND	ND	0.11±0.02^{cd}	0.14±0.02^{ab}	0.14±0.01^{ab}	0.15±0.01^{a}
2-Acetonylcyclopentanone	60415-94-3	$C_8H_{12}O_2$	0.23±0.05^{d}	0.26±0.03^{bcd}	0.26±0.01^{bcd}	0.31±0.01^{ab}	0.29±0.02^{abc}	0.18±0.02^{e}	0.23±0.03^{cd}	0.26±0.06^{abcd}	0.32±0.02^{a}	0.28±0.01^{abcd}
2-Undecanone	112-12-9	$C_{11}H_{22}O$	ND	ND	0.12±0.03^{b}	0.16±0.01^{b}	0.42±0.14^{a}	ND	0.19±0.02^{b}	0.20±0.02^{b}	0.45±0.05^{a}	0.11±0.01^{b}
2-Pyrrolidinone	616-45-5	C_4H_7NO	0.43±0.16^{abc}	0.51±0.11^{abc}	0.49±0.06^{abc}	0.61±0.27^{ab}	0.64±0.23^{a}	0.30±0.05^{c}	0.53±0.11^{abc}	0.40±0.07^{abc}	0.64±0.17^{a}	0.34±0.02^{bc}
**Total**			0.66±0.11^{ef}	0.86±0.13^{de}	0.87±0.08^{de}	1.21±0.29^{bc}	1.34±0.22^{ab}	0.47±0.05^{f}	1.07±0.14^{bcd}	1.00±0.16^{cd}	1.55±0.18^{a}	0.88±0.01^{de}

Sulfides												
Lenthionine	292-46-6	$C_2H_4S_5$	14.96±2.07^{bc}	15.85±2.37^{abc}	10.68±2.88^{c}	18.42±4.16^{ab}	19.19±2.96^{ab}	21.83±5.04^{a}	17.14±3.48^{ab}	15.95±2.33^{abc}	16.19±3.60^{abc}	1.73±0.23^{d}
1,2,4-Trithiolane	289-16-7	$C_2H_4S_3$	18.74±4.41^{e}	103.41±3.06^{a}	71.26±5.03^{cd}	70.16±9.05^{cd}	65.04±2.08^{d}	23.31±2.06^{e}	72.47±4.30^{cd}	63.52±11.01^{d}	79.66±6.69^{c}	92.26±1.75^{b}
2,3,5-Trithiahexane	42474-44-2	$C_3H_8S_3$	ND	0.23±0.02^{cd}	0.38±0.05^{bc}	0.36±0.07^{bcd}	0.21±0.02^{d}	ND	0.49±0.20^{b}	0.38±0.81^{bc}	0.67±0.02^{a}	ND
1,2,4,5-Tetrathiane	291-22-5	$C_2H_4S_4$	19.93±2.12^{a}	12.23±1.71^{b}	13.32±0.18^{b}	14.08±3.17^{b}	16.36±2.39^{a}	2.38±0.42^{d}	13.55±2.98^{b}	13.46±1.63^{b}	12.92±2.33^{b}	6.53±2.73^{c}
1,2,4,6-Tetrathiepane	292-45-5	$C_3H_6S_4$	ND	0.77±0.11^{a}	0.70±0.11^{a}	0.72±0.08^{b}	0.36±0.04^{ab}	ND	0.77±0.08^{a}	0.80±0.07^{a}	0.84±0.09^{a}	0.79±0.15^{a}
Benzyl isothiocyanate	622-78-6	C_8H_7NS	ND	ND	ND	0.39±0.12^{a}	0.50±0.11^{b}	ND	ND	ND	ND	ND
**Total**			53.63±3.29^{d}	132.50±5.10^{a}	96.35±6.98^{c}	104.12 ± 8.08^{bc}	101.66 ± 2.67^{bc}	47.53±7.06^{d}	104.41 ± 8.49^{bc}	94.11±12.24^{c}	110.27±7.57^{b}	101.31 ± 1.71^{bc}
Alcoholas												
1-Octen-3-ol	3391-86-4	$C_8H_{16}O$	0.48±0.13^{e}	1.08±0.19^{abc}	0.88±0.07^{d}	1.00±0.05^{bcd}	0.94±0.10^{cd}	0.44±0.02^{e}	1.11±0.13^{abc}	1.13±0.05^{ab}	1.20±0.05^{a}	1.25±0.07^{a}
2-Ethylhexanol	104-76-7	$C_8H_{18}O$	1.37±0.19^{b}	1.15±0.16^{b}	1.24±0.08^{b}	1.67±0.41^{b}	1.68±0.41^{b}	1.17±0.28^{b}	1.46±0.08^{b}	2.07±1.04^{ab}	2.73±0.81^{a}	1.27±0.11^{b}
Benzyl alcohol	100-51-6	C_7H_8O	ND	ND	ND	ND	16.43±1.80^{a}	ND	ND	ND	8.70±0.83^{b}	ND
Borneol	507-70-0	$C_{10}H_{18}O$	0.59±0.03^{ab}	0.56±0.05^{abc}	0.52±0.02^{bcde}	0.54±0.07^{abcd}	0.55±0.01^{abc}	0.50 ± 0.01^{cde}	0.47±0.02^{de}	0.46±0.02^{e}	0.60±0.06^{a}	0.50±0.02^{cde}
Cedrol	77-53-2	$C_{15}H_{26}O$	0.47±0.08^{ef}	0.81±0.10^{cd}	0.98±0.08b^{cd}	1.11±0.04^{bc}	0.73±0.05^{de}	0.28±0.09^{f}	1.27±0.08^{b}	1.60±0.36^{a}	1.65±0.42^{a}	0.90±0.09^{cd}
**Total**			2.90±0.27^{ef}	3.59±0.44^{def}	3.63±0.09^{def}	4.32±0.53^{cd}	20.33±1.62^{a}	2.40±0.22^{f}	4.30±0.25^{cd}	5.26±0.97^{c}	14.89±1.09^{b}	3.91±0.27^{cde}
Acids												
Acetic acid	64-19-7	$C_2H_4O_2$	0.63±0.22^{ab}	0.63±0.21^{ab}	0.57±0.27^{ab}	0.49±0.15^{ab}	0.63±0.20^{ab}	0.35±0.20^{b}	0.76±0.25^{a}	0.50±0.05^{ab}	0.68±0.22^{ab}	0.63±0.12^{ab}
Benzoic acid	65-85-0	$C_7H_6O_2$	ND	ND	ND	ND	ND	0.56±0.26^{a}	ND	ND	ND	0.10±0.00^{b}
**Total**			0.63±0.22^{a}	0.63±0.21^{a}	0.57±0.27^{a}	0.49±0.15^{a}	0.63±0.20^{a}	0.91±0.42^{a}	0.76±0.25^{a}	0.50±0.05^{a}	0.68±0.22^{a}	0.74±0.13^{a}
Alkenes												
(-)-α-Cedrene	469-61-4	$C_{15}H_{24}$	ND	0.22±0.04^{bc}	0.05±0.00^{e}	0.31±0.04^{a}	ND	0.11±0.06^{d}	0.19±0.01^{c}	0.05±0.01^{e}	0.03±0.01^{ef}	0.24±0.00^{b}
α-Curcumene	644-30-4	$C_{15}H_{22}$	0.23±0.03^{ef}	0.34±0.04^{de}	0.51±0.03^{cd}	0.62±0.06^{c}	0.35±0.05^{de}	ND	0.57±0.09^{cd}	0.88±0.19^{b}	1.14±0.38^{a}	0.40±0.08^{cde}

**Total**			0.23 ±0.03^{e}	0.57±0.07^{cd}	0.56±0.04^{cd}	0.93±0.08^{ab}	0.35±0.05^{de}	0.11±0.06^{e}	0.76±0.10^{bc}	0.93±0.19^{ab}	1.17±0.37^{a}	0.64±0.08^{c}
Others												
1-Acetylimidazole	2466-76-4	$C_5H_6N_2O$	ND	0.47±0.07^{b}	0.48±0.10^{b}	0.65±0.05^{a}	0.46±0.03^{b}	ND	0.41±0.06^{bc}	0.51±0.06^{b}	0.32±0.01^{c}	0.11±0.04^{d}
Benzoxazole	273-53-0	C_7H_5NO	0.38±0.07^{a}	0.21±0.03^{b}	ND	0.20±0.00^{b}	0.24±0.09^{b}	ND	0.08±0.01^{c}	ND	0.05±0.01^{cd}	0.09±0.00^{c}
m-Toluidine	108-44-1	C_7H_9N	ND	0.38±0.03^{ab}	0.40±0.02^{a}	0.39±0.13^{ab}	0.28±0.11^{bc}	0.16±0.05^{d}	0.12±0.03^{d}	0.19±0.02^{cd}	0.19±0.01^{cd}	0.32±0.02^{ab}
Benzyl nitrile	140-29-4	C_8H_7N	ND	ND	ND	0.20±0.03^{b}	0.45±0.04^{a}	ND	ND	ND	0.21±0.04^{b}	ND
Caprolactam	105-60-2	$C_6H_{11}NO$	ND	ND	ND	ND	ND	ND	0.28±0.06^{b}	0.35±0.17^{b}	0.65±0.10^{a}	ND
2,4-Di-tert-butylphenol	96-76-4	$C_{14}H_{22}O$	0.36±0.13^{bc}	0.27±0.03^{c}	0.39±0.11^{bc}	0.40±0.13^{bc}	0.46±0.06^{bc}	0.38±0.14^{bc}	0.42±0.07^{bc}	0.55±0.05^{b}	1.26±0.16^{a}	0.27±0.01^{c}
**Total**			0.74±0.19^{e}	1.32±0.08^{d}	1.26±0.13^{d}	1.84±0.16^{bc}	1.88±0.11^{b}	0.54±0.18^{e}	1.31±0.10^{d}	1.59±0.15^{c}	2.68±0.22^{a}	0.78±0.05^{e}
**Total compounds**			71.41±3.58^{d}	150.23±5.93^{b}	114.97±6.98^{c}	125.64±8.53^{c}	153.91 ± 2.89^{ab}	61.94±6.88^{d}	129.18±9.56^{c}	120.09 ± 14.40^{c}	164.86±7.56^{a}	117.53±3.94^{c}

Different lowercase letters in the same row indicate significant differences ($P < 0.05$). ND: not detected.

Table. S3: Odor threshold and odor activity value (OAV) in the *Lentinus edodes* stipe hydrolysates (LSHs) and control group.

**Compounds**	**Odor thresholds (µg/kg)**	**Odor description**	**OAVs**									
			**Control**	**Flavourzyme**	**Neutrase**	**Papain**	**Bromelain**	**Cellulase**	**FeN**	**FeP**	**FeB**	**FeC**
Aldehydes												
Hexanal	4.5	Sweet, green, fatty, aldehydic	1.35	1.01	1.12	1.44	1.29	0.82	1.12	1.11	1.24	0.92
Heptanal	3	Fresh, aldehydic, fatty, green	<0.1	<0.1	<0.1	<0.1	<0.1	<0.1	<0.1	<0.1	<0.1	<0.1
(E)-2-Heptenal	13	Soap, fat, almond	<0.1	ND	ND	ND	ND	ND	ND	ND	ND	ND
Benzaldehyde	320	Almond, burnt sugar	<0.1	<0.1	<0.1	<0.1	<0.1	<0.1	<0.1	<0.1	<0.1	<0.1

Phenylacetaldehyde	4	Hawthorne, honey, sweet	0.44	0.30	0.27	0.24	ND	0.17	0.27	0.35	ND	0.29
Nonanal	1	Fat, citrus, green	1.11	1.41	1.30	1.18	1.10	2.32	1.01	1.01	1.26	1.29
(E)-2-Nonenal	0.19	Fatty, green, cucumber, aldehydic	1.90	1.05	2.96	2.97	3.70	0.51	1.54	1.20	1.55	1.13
2-Phenylpropenal	/	/	/	/				ND				ND
2,4-Dimethylbenzaldehyde	0.2	/	5.16	5.89	7.11	7.38	9.62	8.11	0.88	1.01	0.85	3.61
Esters												
Ethyl butyrate	1	Apple	ND	ND	ND	ND	9.96	ND	ND	ND	5.04	ND
Isoamyl acetate	2	Banana	ND	ND	ND	ND	0.55	ND	ND	ND	0.35	ND
Allyl hexanoate	500	Sweet, fruity, pineapple	ND	ND	ND	ND	<0.1	ND	ND	ND	<0.1	ND
Benzyl acetate	30	Fresh, boiled vegetables	ND	ND	ND	ND	<0.1	ND	ND	ND	<0.1	ND
Benzyl propionate	/	Flower, sweet	ND	ND	ND	ND	/	ND	ND	ND	/	ND
Butyl benzoate	/	Balsamic, fruity	ND	ND	ND	ND	ND	ND	/	/	/	ND
Tris(2-chloropropyl) phosphate	/	/	ND	ND	ND	ND	ND	ND	/	/	/	ND
Triisobutyl phosphate	/	/	/	ND	ND	ND	ND	ND	ND	ND	ND	ND
2,2,4-Trimethyl-1,3-pentanediol diisobutyrate	0.13	/	1.00	1.14	ND	ND	1.86	1.79	4.27	6.74	6.38	1.77
Ketones												
2-Heptanone	140	Fruity, sweet, coconut, cheesy	ND	<0.1	ND	<0.1	ND	ND	<0.1	<0.1	<0.1	<0.1
2-	/	/	/	/	/	/	/	/	/	/	/	/

Acetonylcyclopentanone												
2-Undecanone	5.5	Fresh, green, orange, rose	ND	ND	<0.1	<0.1	<0.1	ND	<0.1	<0.1	<0.1	<0.1
2-Pyrrolidinone	/	/	/	/	/	/	/	/	/	/	/	/
Sulfides												
Lenthionine	270	Mushroom-like	<0.1	<0.1	<0.1	<0.1	<0.1	<0.1	<0.1	<0.1	<0.1	<0.1
1,2,4-Trithiolane	7.66	Sulfur, onion	2.45	13.50	9.30	9.016	8.49	3.04	9.46	8.29	10.40	12.04
2,3,5-Trithiahexane	0.8	Strong sulfurous onion	ND	0.29	0.48	0.46	0.26	ND	0.61	0.48	0.83	ND
1,2,4,5-Tetrathiane	0.23	Mushroom-like	86.66	53.19	57.91	61.21	71.15	10.37	58.90	58.52	56.15	28.39
1,2,4,6-Tetrathiepane	1.16	Mushroom-like	ND	0.67	0.60	0.62	0.31	ND	0.66	0.69	0.73	0.69
Benzyl isothiocyanate	0.7	/	ND	ND	ND	0.55	0.72	ND	ND	ND	ND	ND
Alcoholas												
1-Octen-3-ol	1.5	Mushroom, earthy, green, oily	0.32	0.72	0.59	0.67	0.63	0.30	0.74	0.75	0.80	0.83
2-Ethylhexanol	300	Rose, green	<0.1	<0.1	<0.1	<0.1	<0.1	<0.1	<0.1	<0.1	<0.1	<0.1
Benzyl alcohol	20000	Sweet, flower	ND	ND	ND	ND	<0.1	ND	ND	ND	<0.1	ND
Borneol	180	Camphor	<0.1	<0.1	<0.1	<0.1	<0.1	<0.1	<0.1	<0.1	<0.1	<0.1
Cedrol	0.5	Cedarwood, sweet, soft	0.94	1.63	1.97	22.22	1.46	0.56	2.53	3.21	3.31	1.79
Acids												
Acetic acid	99000	Acid, fruit, pungent, vinegar	<0.1	<0.1	<0.1	<0.1	<0.1	<0.1	<0.1	<0.1	<0.1	<0.1
Benzoic acid	1000	Balsamic	ND	ND	ND	ND	ND	<0.1	ND	ND	ND	<0.1
Alkenes												
(-)-α-Cedrene	11940	Woody	ND	<0.1	<0.1	<0.1	ND	<0.1	<0.1	<0.1	<0.1	<0.1

α-Curcumene	/	Herb	/	/	/	/	/	ND	/	/	/	/
Others												
1-Acetylimidazole	/	/	ND	/	/	/	/	ND	/	/	/	/
Benzoxazole	/	/	/	0.21±0.03	ND	/	/	ND	/	ND	/	/
m-Toluidine	2000	Aromatic aniline-like	ND	<0.1	<0.1	<0.1	<0.1	<0.1	<0.1	<0.1	<0.1	<0.1
Benzyl nitrile	1200	/	ND	ND	ND	<0.1	<0.1	ND	ND	ND	<0.1	ND
Caprolactam	59700	Amine spicy	ND	ND	ND	ND	ND	ND	<0.1	<0.1	<0.1	ND
2,4-Di-tert-butylphenol	500	/	<0.1	<0.1	<0.1	<0.1	<0.1	<0.1	<0.1	<0.1	<0.1	<0.1

ND: not detected. "/" means no threshold or odor description was found. Threshold in water was cited from the book "*Odor thresholds: Compilations of odor threshold values in air, water and other media*". Odor descriptions come from http://www.flavornet.org/ (accessed on 2 July 2026) and https://pubchem.ncbi.nlm.nih.gov/ (accessed on 2 July 2026).
